# Cortical hot spots and labyrinths: why cortical neuromodulation for episodic migraine with aura should be personalized

**DOI:** 10.3389/fncom.2015.00029

**Published:** 2015-03-05

**Authors:** Markus A. Dahlem, Bernd Schmidt, Ingo Bojak, Sebastian Boie, Frederike Kneer, Nouchine Hadjikhani, Jürgen Kurths

**Affiliations:** ^1^Department of Physics, Humboldt-Universität zu BerlinBerlin, Germany; ^2^Department of Biological Physik, Max Planck Institute for the Physics of Complex SystemsDresden, Germany; ^3^Cybernetics Research Group, School of Systems Engineering, University of ReadingReading, UK; ^4^Department of Mathematics, The University of AucklandAuckland, New Zealand; ^5^Department of Software Engineering and Theoretical Computer Science, Technische Universität BerlinBerlin, Germany; ^6^Martinos Center for Biomedical Imaging, Harvard Medical School, Massachusetts General HospitalCharlestown, MA, USA; ^7^Potsdam Institute for Climate Impact ResearchPotsdam, Germany; ^8^Institute for Complex Systems and Mathematical Biology, University of AberdeenAberdeen, UK

**Keywords:** migraine, reaction-diffusion, spreading depression, gyrification, neuromodulation

## Abstract

Stimulation protocols for medical devices should be rationally designed. For episodic migraine with aura we outline model-based design strategies toward preventive and acute therapies using stereotactic cortical neuromodulation. To this end, we regard a localized spreading depression (SD) wave segment as a central element in migraine pathophysiology. To describe nucleation and propagation features of the SD wave segment, we define the new concepts of cortical hot spots and labyrinths, respectively. In particular, we firstly focus exclusively on curvature-induced dynamical properties by studying a generic reaction-diffusion model of SD on the folded cortical surface. This surface is described with increasing level of details, including finally personalized simulations using patient's magnetic resonance imaging (MRI) scanner readings. At this stage, the only relevant factor that can modulate nucleation and propagation paths is the Gaussian curvature, which has the advantage of being rather readily accessible by MRI. We conclude with discussing further anatomical factors, such as areal, laminar, and cellular heterogeneity, that in addition to and in relation to Gaussian curvature determine the generalized concept of cortical hot spots and labyrinths as target structures for neuromodulation. Our numerical simulations suggest that these target structures are like fingerprints, they are individual features of each migraine sufferer. The goal in the future will be to provide individualized neural tissue simulations. These simulations should predict the clinical data and therefore can also serve as a test bed for exploring stereotactic cortical neuromodulation.

## 1. Introduction

Migraine is characterized by recurrent episodes of head pain, often unilateral, with a prevalence of about 14% in the population, and up to 18% in women (Stovner et al., 2007). In one third of the cases migraine involves additional neurological symptoms, called aura. The neuronal correlate of the aura is spreading depression (SD), a propagating wave of massive disruption in cortical ion and water homeostasis. In the afthermath, SD may also cause the migraine pain by an inflammatory signaling cascade from metabolically stressed neurons to trigeminal afferents in the dura (Karatas et al., 2013).

**Figure 1:**
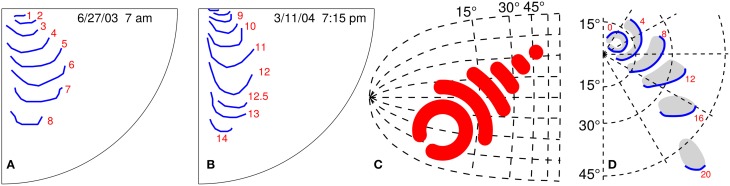
**Empirical and postulated propagating visual field defects. (A,B)** Two similar propagation patterns of self-reported visual field defects during migraine with aura; the attacks were 258 days apart. The red number indicate time in minutes starting from the first recognition of the migraine aura phase. **(C)** Kinematical description of SD wave pattern in the left primary visual cortex (V1). The V1 surface is assumed flat and described by the layout of visual field coordinates. **(D)** Right visual hemifield with the progressive wave pattern that matches the SD pattern in **(C)**. Red number give time in minutes. **(C,D)** are modified from Dahlem and Müller (2003).

Based upon migraine aura symptoms reports like those shown in Figure 1, it has been questioned whether SD spreads concentrically in all directions (Wilkinson, 2004). Instead it was suggested that SD in the human cortex propagates as a localized small wave segment following only one direction (Dahlem and Chronicle, 2004; Dahlem and Müller, 2004; Dahlem and Hadjikhani, 2009). A spatially more restricted SD pattern could result in less symptomatic expression and resolve why such a profound electrophysiological event, which SD clearly is, causes rather minor neurological symptoms (Charles and Baca, 2013). The SD pattern can also be limited to a nonessential area, such that SD might stay silent. Both these factors, taken together, support the assumption that the aura should not be disconnected from the actual headache (Purdy, 2008). If that is the case, there should be an entirely new approach selectively targeting the specific paths of the SD wave segment and thus limiting its noxious effects.

There is strong evidence for localized SD patterns from five different lines of investigation. First, the spatio-temporal development of migraine aura symptoms in the visual field is often localized (Pöppel, 1973; Grüsser, 1995; Wilkinson, 2004; Dahlem and Hadjikhani, 2009; Hansen et al., 2013). This regards retinotopy as a single sensory modality (Figures 1A,B). Second, aura symptoms follow distinct though from attack to attack variable paths when mapped onto the cortical surface across sensory and cognitive modalities (Vincent and Hadjikhani, 2007). Third, we previously suggested a mechanism in a generic mathematical reaction-diffusion model (Dahlem, 2013; Dahlem and Isele, 2013) that can explain the large variety of SD patterns observed (Hansen et al., 2013) as large-amplitude fluctuations that are critically slowed down. This mechanism links the fact that SD patterns in migraine are transient to spatial confinement in the shape of wave segments. This mechanism also proposes the existence of a more global inhibitory signal—likely established by neurovascular coupling—that merely modulates the spread of SD though it can easily be confused for SD in noninvasive imaging depending on blood flow. Fourth, we investigated how localized patterns are influenced and possibly stabilized by the intrinsic curvature of the medium using a torus (Kneer et al., 2014). To a certain degree cut outs of toroidal surfaces can approximate the hills and valleys of cortical gyri and sulci. And finally fifth, we detected localized and also re-entry and other propagation patterns of SD in the gyrencephalic brain using intrinsic optical imaging in animal models (Dahlem et al., 2010; Santos et al., 2014).

These independent lines of evidence are integrated in this study, and further numerical simulations are performed to engage in a new model-based therapeutic brain stimulation approach. The ultimate goal of this approach is (i) to prevent nucleation of SD by spatially targeted lowering of cortical susceptibility, (ii) to shorten the transient decay time of SD, and (iii) to speed up the recovery from the aftermath of SD or even completely suppress long-lasting pain states (i.e., trigeminal or central sensitization). To conceptualize these therapeutic goals, we first describe cortical “hot spots” and “labyrinths” as mainly geometrical configurations that together with the areal, laminar, and cellular heterogeneity of the cortex influence the localized SD pattern formation in the human gyrified cortex in a systematic way. We suggest to consider hot spots and labyrinth paths as target structures in future stereotactic cortical neuromodulation approaches. Our first simulations provide the direction toward large-scale neural tissue simulations as a test bed for exploring the development and quality assurance of such approaches.

## 2. Methods

### 2.1. Mathematical model of wave segments

There is a large set of literature on reaction-diffusion (RD) models in SD (Tuckwell and Miura, 1978; Tuckwell, 1981; Almeida et al., 2004; Miura et al., 2007; Postnov et al., 2009, 2012). In this study, we used a generic model with generic rate functions. The model and its physiological interpretation can be found in a detailed review by Zandt et al. (2015). Briefly, the model is of activator-inhibitor type. The activator *u* describes the front propagation of SD, *u* can diffuse in the medium, and the diffusion coefficient is *D*. The inhibitor *v* is responsible for the recovery phase (spatial confinement in 1D) and for the shape in form of wave segments (spatial confinement in 2D). The latter is achieved by inhibitory global feedback described by the integral term, cf. Krischer and Mikhailov (1994). This term is proportional to surface area of the cortex being invaded by SD multiplied by the coupling strength *k*. The function *H*(·) is the Heaviside step function. This yields

(1) $$\frac{\partial u}{\partial t}\;=u-\frac{1}{3}u^{3}-v+D\nabla^{2}u,$$

(2) $$\frac{\partial v}{\partial t}=\varepsilon \left(u+\beta +k\int H\left(u\right)\text{d}\alpha_{1}\text{d}\alpha_{2}\right).$$

Further parameters are ε and β, the time scale separation between activator *u* and inhibitor *v*, and a threshold parameter, respectively. The independent variables α_1_ and α_2_ are the two spatial coordinates of the cortical surface.

Two remarks about this model are due. First, without inhibitory global feedback (*k* = 0), the RD model still can describe SD propagation as an engulfing ring-shaped wave, or, in the case of an open wave front, as a re-entry wave in the shape of a spiral (Dahlem et al., 2010). Stable localized wave segments do not exist for *k* = 0. Second, for (*k* ≠ 0) stable wave solutions exist in the shape of a wave segment and these solutions are stationary solution in a comoving frame (in other words, the shape is time-independent, they are usually not “breathing” structures). Therefore, it can be concluded that wave segments are also solutions of the system with *k* = 0 for a particular value of β, because the integral term is constant and only adds to the numerical value of β in this case. We rely on this in some of the arguments below. The details have been explained more rigorously and we have to refer the interested reader to these previous studies (Dahlem et al., 2010; Dahlem, 2013; Dahlem and Isele, 2013; Kneer et al., 2014). There, it has been shown that the stable wave segments in systems with *k* ≠ 0 correspond in another system with different β and *k* = 0 to unstable nucleation solutions. Moreover, there are also unstable nucleation solutions for *k* ≠ 0 that evolve into a wave segment when perturbed accordingly. This behavior has also been described in detail in the literature for the model given by Equations (1, 2) and by similar RD systems (Krischer and Mikhailov, 1994; Bode and Purwins, 1995; Schenk et al., 1997).

In order to study the influence of the curvature on the stability of waves segments, the Laplace operator ∇^2^ must be replaced with the Laplace-Beltrami operator Δ_*LB*_ (Davydov et al., 2003) for surfaces given in a coordinate system (α_1_, α_2_):

(3) $$\Delta_{LB}=\sum_{i,j=1}^{2}g^{-\frac{1}{2}}\frac{\partial }{\partial \alpha^{i}}\left(g^{\frac{1}{2}}g^{ij}\frac{\partial }{\partial \alpha^{j}}\right)$$

The nucleation on curved surfaces of this RD model has been introduced by Kneer et al. (2014). Various other RD systems have also been studied in experiments and theory (Maselko and Showalter, 1989; Abramychev et al., 1990; Davydov and Zykov, 1991, 1993; Mikhailov et al., 1994; Davydov et al., 2000a,b, 2002; Manz et al., 2003; Manz and Müller, 2003).

The wave segment pattern in Figure 1C is given by an analytic expression that was derived for kinematic approximation of the RD system for *k* = 0, and its retinotopic representation is shown in Figure 1D, as described by Dahlem and Müller (2003).

### 2.2. Simplified surface construction and simulation

The surface construction of the gross morphology of the calcarine sulcus (CS) has been described by Dahlem and Tusch (2012). The smaller gyral folds and sulci indents that give rise to a random ondulation of this surface have been generated using the software package OpenFOAM (OpenCFD Ltd (ESI Group), 2014). The domain is created by the build-in tool blockMesh. The simulations were performed on a domain which has a local bump centered at the origin and a surrounding region which is flat. The bottom of the domain is given by the rotational symmetric function

(4) $$h_{b}(r)=\{\begin{array}{cc} 20\cos^{2}\left(\frac{\pi r}{40}\right) & r\in [0,20],\\ 0 & \text{else}. \end{array}$$

The top is generated by the constraint of a thickness of one spatial unit. The entire domain has a thickness of one layer and no flux boundary conditions at the top and bottom of the domain. This system can easily be extended to model varying thicknesses, cf. Section 4. The system of equations has a local reaction part and the activator diffuses along the domain. The time derivatives are being discretized by an implicit Crank-Nicholson scheme.

The spatial part is dissected into control volumes leading to a discretization of the computational domain. Inside a control volume the gradient of the flux is approximated using central differencing around the nodal point in the center of the control volume. The control volumes are coupled via the flux between the interfaces of adjacent control volumes. Periodic boundary conditions are applied at the boundary of the domain to mimick an infinitely large domain. However, only single interactions with a local bump were studied. Details of the method are described in Versteeg and Malalasekera (2007).

To generate wave segments the following procedure has been adhered to. For *k* = 0, the model allows for a stable planar pulse propagation in one spatial dimension. An excited initial area (activator equal to one) that spans the entire height of the domain has been generated as initial condition. Such a pulse would spread to the left and right. To break the symmetry the area to the left of the activated area is inhibited (inhibitor variable equal to 0.5). After about 800 time steps a smooth stable pulse propagates to the right. Open ends are created by setting the activator and inhibitor concentration to the equilibrium state at the top and bottom ends of the pulse solution. Only then *k* is set to its nonzero value and the wave segment is stabilized. After about 1000 time steps all transients dynamics or damped out and the stable wave segment propagates to the right. The parameter values used to model the system described by Equations (1, 2) are ε = 0.04, *D* = 1, β = 0.9, and *k* = 0.003. In the last step, the stable wave segment is mapped onto a domain with a bump with varying parameter *d*. The value of the angle ϕ is measured after the wave segment as passed the pump completely.

### 2.3. Matching simulations of migraine aura on individual MRI scans

Anatomically and geometrically accurate representations of the cortex are needed for realistic modeling of SD nucleation and propagation. Furthermore, spatially targeted clinical applications require the extraction of the cortical surface in every individual case, given the considerable variation of the folds and the boundaries of brain areas in the human brain. Structural MRI data can be used to distinguish different tissue types with sufficient spatial resolution, and various open-source and commercial software tools exist for automatic MRI tissue segmentation and mesh generation, including FreeSurfer (Dale et al., 1999; Fischl et al., 1999) and FSL (Smith et al., 2004).

A left V1 surface was obtained by magnetic resonance imaging (MRI) from a migraine sufferer who fulfills the International Headache Society criteria for the diagnosis of migraine with aura, for details see the study by Dahlem and Hadjikhani (2009). We customize and validate the traveling wave patterns of migraine aura pathophysiology by uploading the MRI scanner readings into our self-programmed simulation tool box that uses a C++ software library supporting the creation of finite element codes (Bangerth et al., 2007). The methods involve an automated conversion of 2D triangular mesh from the fMRI into a quadrilateral mesh, for later adaptive refinement and coarsening by the software library.

To generate stable wave segments as an initial condition, we proceeded in a very similar way as described for the scattering simulations at smaller gyral folds and sulci indents above. The parameter values used to model the system described by Equations (1, 2) are ε = 0.04, *D* = 1, β = 1.2, and *k* = 0.0045. Note the change in β and *k* compared to the simulation on the surface with a bump. These different values select a solution of a wave segment with different size to better match the aura symptoms. The main difference in this case is that we have to project this stable solution from a flat surface onto the intrinsically curved V1 surface. There is not a straightforward way to calculate a natural initial condition, because there is no stationary shape on a surface with varying curvature. Even on a flat cortex the natural initial conditions (conditions that ignite SD in the cortex) probably correspond to random spatial patterns caused by some large fluctuation in either metabolic energy supply or neural activity or in both. We addressed this problem using cortical feature maps in V1 (Dahlem and Isele, 2013). For our simulations here, the initial conditions had to satisfy the correct initial propagation direction to match the visual field defects shown in Figures 1A,B, which was drawn from the visual field defects as described by Hansen et al. (2013).

### 2.4. Gaussian curvature *K* for a discretised cortical surface

To determine the Gaussian curvature, we use a cortical surface originally extracted with CIVET (Kim et al., 2005). This surface consists of 163,840 triangles with 81,924 vertices in two separate closed hemispheres, where each of the mesh vertices can be considered as representing the surrounding neural tissue and its connectivity (Bojak et al., 2010, 2011).

A variety of methods have been proposed for the estimation of curvature from such discretised mesh surfaces, see the review of Magid et al. (2007). An extensive investigation of these methods is beyond the scope of this article. Magid et al. (2007) identify fitting osculating paraboloids as the method of choice if one wants to employ only one method, due to its good stability, convergence and accuracy. This method also has the advantage of conceptional simplicity and computational speed. Essentially, to compute the curvatures at a specific vertex, its coordinates and those of its immediate neighbors are transformed by translation and rotation so that the vertex is now located at the origin and the surface normal at the target vertex coalesces with the *z*-axis. This surface normal can be estimated as the average of the normals of the triangles that the vertex forms with its neighbors. Then a least square fit of the canonical parabolic form *z* = *ax*^2^ + *bxy* + *cy*^2^ to the vertex and its surrounding neighbors is performed, making the resulting paraboloid optimally osculate, i.e., be as near as possible to all these vertices. From the resulting fit values the mean *H* = *a* + *c* and Gaussian *K* = 4*ac* − *b*^2^ curvatures, respectively, can be obtained directly. If needed, the principal curvatures $\kappa_{1,2}=H\pm \sqrt{H^{2}-K}$ are easily computed from these values. Overall, the method hence consists in estimating the curvatures at a mesh point by identifying them with the curvatures of a locally fitted paraboloid.

In practice, mesh surfaces obtained from MRI tissue segmentation are uneven at the scales of the original voxels, since the tissue interfaces may pass through a voxel at various levels and angles, and in addition can suffer from overfitting leading to artificial ripples in the extracted surfaces. To guard against overestimating the curvature due to such artifacts, we have employed the following procedure here: We estimate the curvature not only from the vertex and its surrounding ring of nearest neighbors (yielding *H* and *K*), but also from the same vertex and the surrounding ring of next-to-nearest neighbors (yielding $\tilde{H}$ and $\tilde{K}$), i.e, from target vertex and the ring formed by the neighbors of the neighbors of the target vertex, where that ring obviously excludes the target vertex itself. We now consider the difference of these estimates δ*H* = *H* − $\tilde{H}$ and δ*K* = *K* − $\tilde{K}$, respectively, across all *n* = 81,924 vertices and use Chauvenet's criterion to detect outliers in these differences: Given the mean μ_δ*H*_ and standard deviation σ_δ*H*_ of the δ*H*, we consider as outliers those points where *n* · erfc(|δ*H* − μ_δ*H*_|/σ_δ*H*_) < 0.5. At regular points we use the *H*, whereas at outlier points we use whichever value is smaller, *H* or $\tilde{H}$. The same procedure is used to determine whether *K* or $\tilde{K}$ should be used. This method provides a conservative estimate of the curvature suppressing local artifacts. For the particular surface used here, 1.4% of vertices were outliers in *H*, and 0.8% in *K*, thus the surface for the most part proved to be smooth.

In **Figure 5** we show the result for our cortical surface. The actual range of values is − 2.343/mm ≤ *K* ≤ 2.429/mm. However, to enhance the visibility of the mostly small curvature of cortex, we show $\kappa_{\text{geo}}\equiv \text{sign}(K)\sqrt{\left|K\right|}$ on a color scale [− 1.56/mm, 1.56/mm], as indicated by the color bar. κ_geo_ is basically the *geometric* mean of the principal curvatures κ_1_ and κ_2_, and the Gaussian curvature *K* = sign (κ_geo_)κ^2^_geo_. From right to left in **Figure 5**, we zoom in on the spot with largest negative Gaussian curvature *K* = − 2.343/mm in two steps, as indicated by the orange and yellow boxes, respectively. One can clearly see that this potential nucleation point is buried deep in a sulcus of the visual cortex. We discuss these results further below.

## 3. Results

### 3.1. Cortical hot spots and principal effects of Gaussian curvature

The principal idea of cortical “hot spots” and paths that span cortical “labyrinths” is firstly illustrated in a simplified way. We describe them as anatomical landmarks with a particular geometrical configuration. We separately take into account the gross morphology of the calcarine sulcus (CS) and smaller gyral folds and sulci indents that give rise to a random ondulation of the CS surface.

#### 3.1.1. Gross morphology of the calcarine sulcus

The CS is a reliable landmark of the primary visual cortex (V1). Visual migraine auras often originate in V1 or in higher visual areas neighboring V1 (Figure 1). Therefore, CS can be considered a typical fold in which many migraine auras originate and travel through.

**Figure 2:**
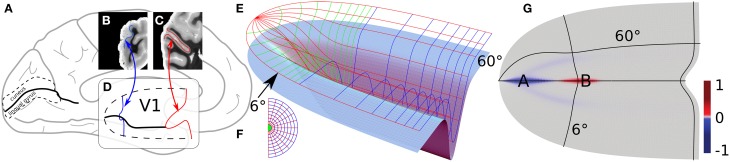
**Principal folding pattern correlating with a cortical area defined by a functional field**. Modified from study by Dahlem and Tusch (2012). **(A)** Medial side of the brain. One the occipital pole to the left, the calcarine sulcus is marked (think black line). **(B,C)** Coronal section through the occipital pole. Blue and red line traces the stria of Gennari as a marker of the primary visual cortex (V1), which is outlined in **(A)** by a dashed line. **(D)** Gross 3D shape of V1, about two-thirds of the surface area lie within the CS walls. The ventral and dorsal half of V1 are assumed to be symmetric with respect to the fundus, in particular, they extend equally long into the anterior direction, the shape of the walls are approximated with smooth profiles (see study by Dahlem and Tusch (2012) for details). **(E)** Light blue surface as in **(D)** with the retinotopic grid on top. **(F)** Visual field polar coordinates. **(G)** Gaussian curvature of V1.

CS is a cortical fold formed by the cuneus and lingual gyrus on the medial surface and runs forward to the corpus callosum (Figure 1A). The fundus of CS is, roughly speaking, the curve of maximum depth that then further spans the length of CS. The surface area of V1, which lies in CS, can, for example, easily be identified post-mortem by the stria of Gennari, i.e., a band of myelinated axons (see Figures 2B,C).

The dorsal and ventral banks of CS can be heavily ramified with smaller gyral folds and sulci indents. To illustrate the main folding pattern with respect to the visual field coordinates (Figures 2E,F), the CS surface has been streamlined (Dahlem and Tusch, 2012). A real example is considered in detail in Section 3.2. On the streamlined model surface (light blue in Figure 2E) the entrance of CS is a saddle surface. It has negative Gaussian curvature (blue, see Figure 2G). Farther down CS, when the fundus almost reaches its maximum depth, the Gaussian curvature becomes positive (red). Elsewhere, the Gaussian curvature is very close to zero or zero (gray).

We suggest to consider regions with large absolute values of negative Gaussian curvature, such as at point A in Figure 2G, as cortical hot spots of SD generation. This follows from previous studies that showed that negative Gaussian curvature causes a smaller nucleation barrier for reaction diffusion waves such as SD (Kneer et al., 2014). Therefore, these areas can more easily lead to subsequent autocatalytic growth of seeds caused by the spatio-temporal fluctuation in homeostatic control.

We also showed that unstable nucleation solutions in a flat medium can be stabilized by the negative gradient of Gaussian curvature on the outside of a torus (Kneer et al., 2014). This provides an alternative mechanism of wave segment creation. A surface similar to the outside of a torus is assumed when the fundus almost reaches the maximum depth of CS at point B in Figure 2G. Note that gyral crowns are also of similar shape and the Gaussian curvature does not distinguish between gyrus and sulcus (cf. Section 4).

#### 3.1.2. Single sulci indent

Before we consider in detail the influence of randomly changing negative and positive Gaussian curvature in real cortical surfaces, we describe the influence of a single sulci indent on an otherwise flat surface. The flat surface can be thought of as either the dorsal or ventral bank of CS. Indents have rather small in extent (diameter) but relatively large altitude. Such structures are not unsusal, in particular in the deep sulci of the occipital pole.

**Figure 3:**
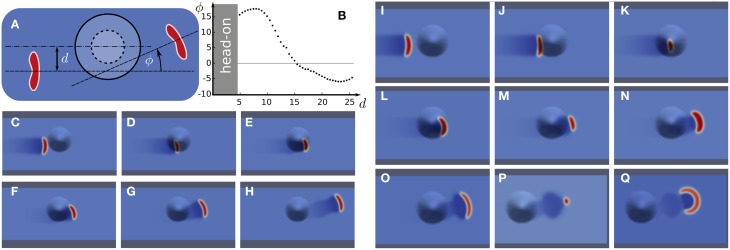
**Wave segments deflected by a bump. (A)** Scheme with definitions of offset parameter *d* and deflection angle ϕ. **(B)** Deflection angle ϕ vs. offset *d*. **(C–H)** Numerical simulation of a positive deflection. **(I–Q)** Numerical simulation of head-on collision with wave destruction.

In Figure 3, we show the scattering effect of a local bump, representing a small sulci indent, on a traveling stable wave segment. Whether stable wave segments were deflected by positive or negative angles ϕ (Figure 3A) depends in a systematic way on the offset *d* (Figure 3B). In the example shown in Figures 3C–H the stable wave segment is deflected by a positive angle ϕ. This can intuitively be understood. The near side of the wave segment, with respect to the pump location, has to travel a longer distance and therefore falls behind. The net effect is then a turn toward the bump indicated by a positive angle ϕ. This simple explanation, however, is only correct to a medium rage of *d* between value 5 and 15.

For a direct collision (*d* = 0) or too small an offset (*d* < 5), the stable wave segment can even get eradicated (Figures 3I-Q). We call a wave-bump interaction in this range, which eradicates the wave segment, a head-on collision. For example, the wave disappears a few time steps after the snapshot shown in Figure 3Q. A bump always causes first a negative Gaussian curvature and then the sign of the Gaussian curvature changes (indicated by the dashed line Figure 3A) and the bump surface assumes a positive Gaussian curvature. The critical effect that causes the wave segment to vanish is due to large size and shape variations. These in turn are due to the rapidly changing gradients in Gaussian curvature in a head-on collision. Note that the size variations continue long after the bump is passed. This oscillation is called “breathing”. It indicates that wave segment is a stable focus in phase space (Strogatz, 1994). The perturbation in a head-on collision, however, has kicked the system out of the basin of attraction of the stable focus. For much smaller bumps (lower altitude) interactions even for *d* = 0 will only cause perturbation inside the basin of attraction and therefore not eradicate the wave segment. These would consequently then not be called a head-on collision, because the segment survives unchanged though it performs damped breathing.

Let us briefly describe the mechanism that actually destroys the wave outside the basin of attraction. For too large an amplitude of the subsequent breathing, propagation is not possible anymore because the wave size gets too large. The wave size shown in Figure 3Q is a last deep gasp close to the final frontier called propagation boundary, see the review by Zandt et al. (2015) and references therein for details. Briefly, a large wave size leads to increased threshold β by global inhibitory feedback described in Equation (2). Above this well-defined value of the threshold the propagation becomes impossible. The global inhibitory feedback could stem from dilations of surface vessels in regions that show no directly increased neural activity (Gao et al., 2014) and thus have higher resistance against SD invasion (cf. Section 4).

For large offsets *d*, the deflection can be by negative angles ϕ (Figure 3B). This can also intuitively be understood. Only the tip at the open end experiences the geometrical change. This change is by a negative Gaussian curvature. Due to the diminished surface area at negative Gaussian curvature, with respect to a flat surface, the tip can more quickly recruit the tissue. This is because in front of the tip the surface area is reduced and by an effective convergence in diffusion this tip grows more than the one on the opposite end. As a result, the wave is deflected by negative angles ϕ. The same effect also causes the smaller nucleation barrier at the location of hot spots.

### 3.2. Cortical labyrinths

To introduce the idea of cortical labyrinths, we use real cortical surfaces. First, we consider the cortical area V1 and a simulation of a stable wave segment on this surface. Next, we consider the whole cortical surface with its heterogeneous distribution of Gaussian curvature.

#### 3.2.1. Individual path in V1

**Figure 4:**
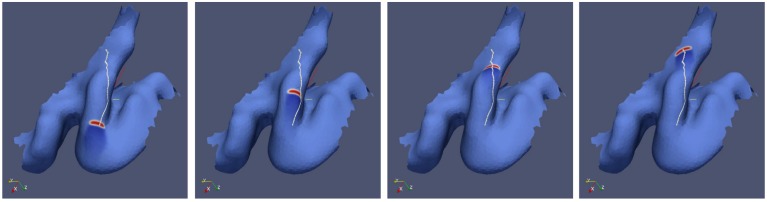
**Simulation of spreading depression on an individually shaped cortical surface obtained by MRI from a migraine sufferer**.

A left V1 surface was obtained by magnetic resonance imaging (MRI), see Section 2. On this surface we simulate the path a stable wave segment takes (Figure 4). We placed the wave segment near the occipital pole pointing toward the direction of the cunes (cf. Figure 2A). The propagation pattern of visual aura, which we match with this simulation, is located within the lower quadrant of the right visual hemifield near the vertical meridian, see Figures 1A,B. The lower quadrants of visual hemifield are mapped onto the ventral bank of CS and the cuneus, on which the vertical meridian is also represented. The initial position was estimated as the location of the neural representation of the fovea, which is located at the occipital pole often extending about 10mm onto the lateral convexity.

The path the simulated wave segment takes matches closely the path of the visual aura in the visual field within the first 8 min of an attack at one day and within minutes 7–14 at another day (Figures 1A,B). During these periods, it propagated within the lower visual quadrant in the right visual hemisphere from the center to the periphery and, at about 8° eccentricity, it disappeared or was clinically silent for 7 min and reappeared (Dahlem and Hadjikhani, 2009). The anatomical and physiological features of this distinctive and further aura patterns of this migraine sufferer were also investigated by Hansen et al. (2013). Let us remark that because SD propagates with about 3 mm/min, 8 min correspond to a distance of about 24 mm, which is about the size of the particular gyral crown on the cuneus (Figure 4).

After we simulate the individual path in V1 of this distinctive aura attack, we can determine the start and end point. These points where given by the center of gravity of the wave segment at the respective positions. Between the start and end point, we also calculate the shortest distance path (white line in Figure 4). It can be seen that the actual path taken by the simulated wave segment overlaps by and large with the shortest distance path.

The idea of the shortest path can be misleading as the wave segment does not teleologically propagate to its final position. Geodesics are locally the shortest path between points in the space, but they are not necessarily the shortest path globally. An exclusively local criteria for the wave propagation is that the osculating plane of the geodesic line is perpendicular to the surface tangent plane. This criteria is probably the reason why wave segments approximately follow with their center of gravity geodesics. Yet, as shown in Section 3.1.2, wave segments can be deflected by small but high gyral folds and sulci indents. Such geometric obstacles in the path of a wave segment may not interfere with the center of gravity of the wave segment. Hence, wave segments will not exactly travels along geodesics that are defined by the center of gravity alone.

#### 3.2.2. Distribution of Gaussian curvature and other heterogeneities

**Figure 5:**
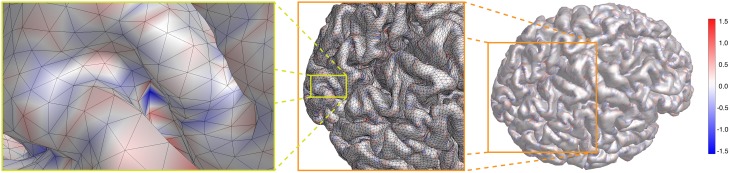
**For a cortical surface $\kappa_{\text{geo}}\equiv \text{sign}(K)\sqrt{\left|K\right|}$ is shown on a color scale [− 1.56/mm, 1.56/mm], as indicated by the color bar**. From right to left, the spot with largest negative Gaussian curvature *K* = − 2.343/mm is brought into focus by zooming closer twice, as indicated by the orange and yellow lines, respectively. It represents a potential nucleation point for SD.

Last, we calculate the distribution of the Gaussian curvature of a whole cortical surface (Figure 5). It can be seen that the curvature overall deviates only moderately from zero. Larger indents can be, however, in the deep sulci of the occipital pole.

It is well known that the cerebral cortex also exhibits considerable regional heterogeneity from cellular, to laminar and areal heterogeneity as briefly outlined in the discussion. Hence, it is important to estimate spatial scales of the heterogeneous distribution of Gaussian curvature in comparison. The change of sign occurs every couple of millimeters, although lager domains and connected stripes of one polarity (negative or positive value of Gaussian curvature) exist.

Ignoring for the moment any other heterogeneity, even the set of permitted geodesics starting from a given point is a complex network. Non-permitted geodesics are those that lead into dead ends due to head-on collision with unfavorable curvature conditions (in addition to other unfavorable heterogeneities, which we have for the moment ruled out, but see Section Conclusion). There can be several geodesics on the cortical surface between two given points. So, in principle, geodesics that start from the some point can later cross again (Maekawa, 1996). A geodesic is, of course, conceptually similar to a straight line in a flat geometry. Given a common start point, straight paths in a flat surface simply span radially lines. Hence curvature makes a significant contribution to the traveling paths. They span a labyrinth through which wave segments can travel. This labyrinth exists even in the absence of other heterogeneities, which are probably at least as relevant and will further modify the structure of the labyrinth.

To define the “cortical labyrinths,” we have to take into account the geodesics and further effects of the Gaussian curvature on the traveling wave segment (see Section 3.1.2, deflection and head-on collisions Figure 3) as well as regional cellular, laminar, and areal heterogeneity. Without measuring or defining all the various structures and make them amenable to numerical simulations, we can always fall back to an operational definition by principle. We call a labyrinth the set of paths a wave segment with a given size can take starting form a particular location on the cortex. To calculate this is computationally expensive as labyrinths depend on the starting point and wave size (defined in flat geometry). Moreover, we currently cannot include information on regional heterogeneity in necessary detail (see Section 4).

As this is beyond the scope of this study, let us summarize and highlight the similarity to a labyrinth structure. Wave segments can only travel on fixed paths. Some of these paths will be a dead end. The random ondulation to the gross cortical morphology alone will already render the particular labyrinths an individual feature, like a fingerprint, of each migraine sufferer.

## 4. Conclusion

### 4.1. Regional heterogeneity in the cortex

The cerebral cortex exhibits regional heterogeneity. This not only can considerably influence the path an SD segments takes, it also actually reveals the only information we currently have about these particular paths, namely by patient's symptom reports. For both reasons, the parcelation into cortical areas as functional domains should be stressed among various other factors that introduce heterogeneity in the cortex.

#### 4.1.1. Clinical manifestations of SD wave segments

It seems that a localized SD wave segment travels through several different cortical areas along a coordinated path in a single migraine aura attack—though from attack to attack different paths may be taken. This is inferred from the fact that migraine aura symptoms frequently occur in patterns each fitting a particular spatially aligned distribution of various successively impaired cortical functions (Vincent and Hadjikhani, 2007).

A full-scale attack is characterized according to the Headache Classification Committee of the International Headache Society by multiple sensory and/or cognitive symptoms during the aura phase. Each individual aura symptom can last up to 60 min, thus the acceptable maximal duration for three different symptoms is 180 min or a path length of 54 cm. This seems reasonable for a narrow wave segment meandering in an area of ~2000 cm^2^ folded and ondulated cortical surface. A segment of 0.5 cm width covers only ~1.35% of the available surface area. In contrast, an engulfing SD with a radius of 54 cm would invade 3.5 times more surface area than is actually available.

#### 4.1.2. Functional domains and anatomical landmarks

Cortical areas are defined based upon cytoarchitecture, functional studies, and subcortical connectivity. These factors can be independent. In the visual cortex, for example, except for V1, there is no correspondence between cytoarchitecture (Brodman) and function (defined by retinotopic mapping). Of course all these heterogeneities need to be considered in addition and in relation to Gaussian curvature when trying to estimate the set of preferred paths of SD propagation. Moreover, sensory and motoric domains can have some relationship to the gross sulcal and gyral morphology. These factors are not independent. In particular, subcortical connectivity and sulcal and gyral morphology could be related by developmental mechanics (Hilgetag and Barbas, 2005). Yet, there also is substantial interindividual variability in both the size and location (Rajkowska and Goldman-Rakic, 1995; Thompson et al., 1996; Roland and Zilles, 1998; Amunts et al., 1999).

Only in a few cases more or less precise correlations between the folding pattern and functional domains have been demonstrated. The most reliable relation is the calcarine sulcus (CS, see Figure 2) as a landmark of the primary visual cortex (V1) (Stensaas et al., 1974; Gilissen and Zilles, 1996; Andrews et al., 1997). For visual areas outside V1, a purely anatomical identification is also quite reliable for V5, which lies at the intersection of the ascending limb of the inferior temporal sulcus and the lateral occipital sulcus (Watson et al., 1993; Walters et al., 2003). The primary auditory cortex has a clear spatial relationship with Heschl's gyrus (Gaschler-Markefski et al., 1997; Rademacher et al., 2001). All these functional domains are relevant in relation to migraine aura symptoms. Last, the motor cortex can be identified by the position of the central sulcus (Lotze et al., 2000). While this is often suggested as a barrier for an engulfing SD pattern, there is evidence that also the prefrontal cortex can be affected (Schipper et al., 2012; Borsook et al., 2014).

#### 4.1.3. Curvature, folding, and thickness

An important fact is that with the cortical folding pattern the cytoarchitecture also varies in a systematic way. There is a crucial distinction to be made. The folding pattern is mainly determined by extrinsic curvature, while Gaussian curvature is intrinsic curvature. Simply said, the SD wave as an “inhabitant” of the cortical surface feels only intrinsic curvature directly. Extrinsic curvature can only indirectly, that is, via the cytoarchitecture, influence SD propagation. In other words, the effect by Gaussian curvature and by differences between gyral vs. sucal convolutions are distinctively different.

Gyral convolutions of the cerebral cortex are thicker than sulcal ones, which is specifically due to an increase in the thickness of the deep layers (V+VI). The increased neuron number and increased thickness of these deep gyral layers correlate so that the density of deep layers is relatively invariant across the cortical landscape, see study by Hilgetag and Barbas (2005) and references therein. If the cell density is constant in different thicknesses of deep layers, volume fraction between intra- and extracellular space and volume-to-surface-area relation should also be invariant. These factors determine important electropyhsiological properties of SD on a cellular level (Hübel and Dahlem, 2014; Hübel et al., 2014).

Notwithstanding, cortical thickness differences have been reported in migraine sufferers in two studies (Granziera et al., 2006; DaSilva et al., 2007) and the possible implications have been discussed throughly by Hadjikhani (2008), so that we can refer the interested reader for details to this. Briefly, focal dysplasias may render the cortex more excitable, which could then add to the effect of negative Gaussian curvature to create hot spots. The cortical thickness changes can be also a result of plasticity following repetitive episodes of pain, which may suggest that there could also be protective changes in plasticity following repetitive neuromodulation.

The central point taken away from these studies on natural and abnormal variations in cortical thickness for the current study is that the approximation of the cortical sheet as a 2D surface does not hold in all circumstances and that the problem can ultimately only be resolved in a neural tissue simulation that extends the requirements and constraints of circuit simulation methods on a cortical sheet (in the continuum limit neural fields) by creating a tissue coordinate system that allow geometrical analysis (Kozloski and Wagner, 2011).

#### 4.1.4. Cortical grade separation

Another noteworthy heterogeneity concerns the laminar structure. Studies in rat show that cortical DC shifts associated with SD can move either in layers near the cortical surface or in deeper cortical layers below a border zone between layer IV and layer V (Richter and Lehmenkühler, 1993). Layer IV is the granular layer of the cortex which could have for SD an unfavorable high volume-to-surface-area. There is not always a strong correlation between the time course of DC shifts in upper and lower cortical structures. It has been suggested that in human, the occurrence of SD in upper and/or deeper cortical layers could have implications on the type of the migraine attack (aura with headache, aura without headache or headache without aura) (Richter and Lehmenkühler, 1993). Grade separation would add significantly to the complexity of a labyrinth structure as at-grade intersection of a non-or-all phenomenon such as SD would cause annihilation or merging but not bypassing cross each other. Much of the anterior cortex including the prefrontal cortex, however, lacks a defined layer IV, thus the labyrinth is at-grade in an agranular cortex.

#### 4.1.5. Vasculature

SD does not only cause a hemodynamic responses (Dreier, 2011). SD can also be caused by hemodynamic abnormalities and its propagation path can be changed by hemodynamic action. For example, large and correlated fluctuations originating from autonomic subnetworks have been suggested to occur as part of a typical tipping point behavior in the prodromal phase of migraine (Dahlem et al., 2014). These global fluctuations can ignite SD locally in a hot spot by crossing there the nucleation threshold for propagation first.

Pial vessels, which distribute blood from the cerebral arteries to the penetrating arterioles, can locally block the propagation (Santos et al., 2014). The penetrating arterioles occur about every 300μm and the pial surface artery network is an interconnected honeycomb-like mesh (Blinder et al., 2010). The regulation of cerebrovascular tone is coordinated by perivascular nerves, glia, and blood pressure and its hydraulic functions. Vessel dilations can propagate into this pial mesh also mediated by fast electrical connections between endothelial cells. In fact, surface vessels show much larger dilations than their intracortical branches and this dialation can also occur in regions that show no directly increased neural activity but are neighboring regions that do show increased activity (Gao et al., 2014). This may provide a fast and long-range inhibitory feedback, as described by Equation (2).

It is also noteworthy that with laser speckle technology the cerebral blood flow was measured in patients with malignant hemispheric stroke after hemicraniectomy. The path of SD was visualized and this was also suggestive of a small wave segment form (Woitzik et al., 2013).

### 4.2. Hot spots and labyrinth paths as target structures in stereotactic neuromodulation approaches

Various treatment strategies for migraine are available today. It cannot be stressed enough that the recommended approach based upon clinical experience is an integrated headache care. Treatment of acute migraine attacks and prophylaxis is accompanied by education and counseling, behavioral therapy and treatment of co-morbidities. However, with respect to drug treatment “acute treatment and preventive therapy of migraine seems to have come to a halt recently. The last real innovation in the treatment of migraine attacks were the triptans, which were introduced 20 years ago. […] [I]t is time to explore new avenues in the treatment of migraine” (Diener, 2013).

**Figure 6:**
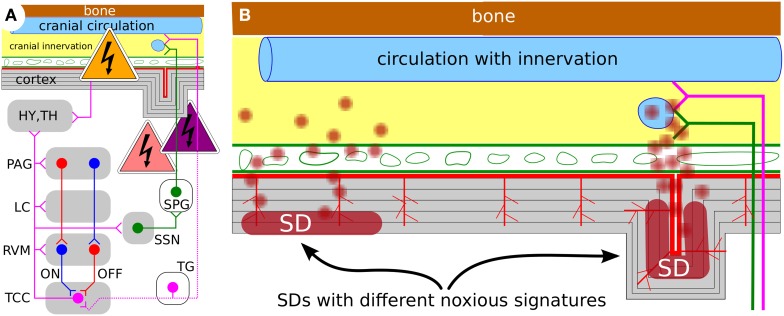
**(A)** Working model ot the migraine-generator network with accessible intervention points (see text) (Dahlem, 2013). **(B)** Schematic representation of a cortical cross section with meninges and skull; not to scale. The effect of SD and its noxious signature could significantly vary with overall size of affected area and SD's gyral, sulcal, and laminar location. Future studies will be needed to design specific treatment stimulation protocols that take into account the noxious signature and its phase within the migraine cycle.

Computational models of migraine not only help to better understand this disease. They also help to identify new opportunities to intervene. Our results suggest that the primary objective in research relating SD to migraine pain should be directed to obtain a measure of the different noxious signatures that are transmitted into the meninges and drive the migraine-generator network into the pain state, i.e., central sensitization (Figure 6). In this context, neuromodulation is of particular interest, because computational models can identify the accessible intervention points. For example, for the treatment of chronic migraine (>15 headache days per month) with transcranial direct current stimulation (tDCS) the current flow through subcortical brain regions associated with pain perception and pain modulation (i.e., trigeminocervical complex (TCC), rostral ventromedial medulla (RVM), locus coeruleus (LC), and periaqueductal gray (PAG), see Figure 6A) was estimated using a computational model (Dasilva et al., 2012).

In analogy to pharmaceutical substances, specific stimulation protocols for medical devices should be approached by rational design, in particular with respect to the various pain phase of the migraine cycle. The goals are also shared with drug treatment, namely to (i) achieve fewer attacks, (ii) abort attacks at an early stage, e.g., in the prodromal or aura phase, and (iii) lessen the severity in the pain phase. To achieve these three goals our results suggests framework conditions on three strategies toward one preventive and two acute therapies with stereotactic neuromodulation for episodic migraine, such as tDCS, transcranial alternate current stimulation (tACS), and transcranial magnetic stimulation (TMS).

- To achieve fewer attacks, our results suggest that one must first identify individual cortical hot spots. Targeting hot spots selectively minimizes intervention. This seems advised because the appropriate stimulation protocols have to cause lasting plastic alterations of cortical excitability. The rational is to lower susceptibility at individual hot spots to prevent nucleation of SD. This can be done with image-guided TMS sessions during the attack-free interval. The intervention probably even must be done outside ictal phase, because protocols may focally increase neural activity to induce lasting protective preconditioning-like effects that would bear negative impact in the acute phase.
- To abort attacks in the aura phase is the current rational of portable TMS devices; patients were instructed to begin treatment as soon as possible after aura symptoms where noticed and always within 1 h of aura onset (Lipton et al., 2010). In the period after SD is ignited, previous theoretical model studies proposed that the inevitable decay of the cortical patterns is critically slowed down by a bottle-neck situation (Dahlem et al., 2013; Dahlem and Isele, 2013). Our results here suggest that these patterns are both selected and temporally stabilized by labyrinth paths. Taken together, the current two single pulse paradigm (for ~0.5, ~30 s apart) are likely to turn with a certain success rate wave segments into faster-decay paths or dead ends. Repetitive TMS with low amplitude, however, could be more effectively shorten the transient decay time. Noise during a bottle-neck passage (Strogatz, 1994) induces large fluctuations allowing the dynamical system to explore nearby states and faster traverse the bottle-neck passages in phase space.
- To speed up the recovery from the aftermath of SD or completely suppress a long-lasting pain state (i.e., trigeminal or central sensitization) the stimulation protocol need to intervene with the inflammatory signaling cascade from metabolically stressed neurons to trigeminal afferents in the dura (Karatas et al., 2013). Our results suggest that the cascade from the brain paremchyma to the dura is effected by geometrical constraints (Figure 6B). For example, if the labyrinth path is entirely in a sculus, sufficient concentrations of inflammatory mediators can be siphoned from the perivascular space into the subarachnoid space. One must first identify the individual cortical labyrinths to identify optimal target regions where the inflammatory cascade can be interrupted. In this context it is interesting to note that despite the gyral crown of the cortex being subjected to a larger magnetic field magnitude during TMS, the sulcal banks show larger cerebral blood flow responses (Krieg et al., 2013).

Strategies (ii) and (iii) describe early and on-demand interventions during the pre-ictal state. Aura symptoms can announce the pre-ictal state in about 30% of the cases. Yet these are not the only indicators of the pre-ictal state. Also other sensory, affective, autonomic, and cognitive functions are altered in this phase and provide means by which migraine suffers become aware of pre-ictal “errors” (Borsook et al., 2014). Strategy (i) seems more bold, as we suggest that migraine might be unlearned. Identifing cortical hot spots and cause plastic changes at these foci is but only first step toward electroceuticals (Famm et al., 2013) that are currently discussed to override pain signals or reprogram circuits from accessible intervention points (Figure 6A).

Already, the first transcutaneous electrical nerve stimulation (TENS) device and the first TMS device were FDA-approved for episodic migraine for prevention and during the aura phase, respectively. Other neuromodulation including invasive methods are approved only for chronic migraine. Focused ultrasound that can open the blood brain barrier in targeted regions is also discussed (Bing et al., 2014) and would provide means to target hot spots and labyrinth paths selectively by local delivery of drugs. If the next generation of noninvasive devices due to model-based optimization of stimulations protocols has less adverse effects and becomes more efficient, patient groups with high frequency episodic migraine attacks and unsatisfactory treatment response will certainly be open to such a treatment.

The cortical target structures that we suggest based on computer simulations of SD are like fingerprints, they are individual features of each migraine sufferer. We use a model-based approach to explain why cortical neuromodulation for episodic migraine should be personalized. The next step will be to provide individualized neural tissue simulations delivering the same output as clinical data. This can then serve as a test bed for further exploring the use of personalized stereotactic cortical neuromodulation.

### Conflict of interest statement

MD has consulted for eNeura Inc in 2012. The authors declare that the research was conducted in the absence of any commercial or financial relationships that could be construed as a potential conflict of interest.
